# Seroprevalence and risk factors of bovine respiratory syncytial virus in cattle in the Nineveh Governorate, Iraq

**DOI:** 10.14202/vetworld.2019.1862-1865

**Published:** 2019-11-27

**Authors:** Khder Jassiem Hussain, Maab Ibrahim Al-Farwachi, Sadam Dhahir Hassan

**Affiliations:** Department of Internal and Preventive Medicine, College of Veterinary Medicine, University of Mosul, Mosul, Iraq

**Keywords:** bovine, pneumonia, prevalence, respiratory syncytial virus

## Abstract

**Background and Aim:**

Bovine respiratory syncytial virus (BRSV) is one of the main causes of severe pneumonia, interstitial edema, and emphysema in cattle. The current study investigated the prevalence and risk factors of BRSV in cattle in the Nineveh Province, Iraq.

**Materials and Methods:**

Between September 2017 and September 2018, 450 serum samples were collected from non-vaccinated cattle of different ages and breeds for BRSV testing. The epidemiological information of the animals was recorded. The prevalence of the disease was determined using an indirect enzyme-linked immunosorbent assay kit.

**Results:**

The prevalence of BRSV was 83.11%, and it was significantly (p<0.05) higher in cattle aged greater than 7 months-1.5 years than in older animals; in imported cattle than in Native animals; and in animals originating from large herds (100 animals) than in those from smaller herds (40 animals). There was no significant difference between BRSV prevalence in male and female animals. When samples from different regions of the Nineveh Governorate were compared, the northern region was associated with the highest prevalence of the disease. Samples harvested in the winter displayed the highest BRSV titer, compared to those collected during the other seasons.

**Conclusion:**

BRSV is prevalent in cattle from the Nineveh Governorate. Risk factors such as animal age, origin, herd size, and the herd’s geographical location are associated with an increased prevalence of the disease in this region. Routine vaccination programs should be adopted to reduce the prevalence of BRSV.

## Introduction

Bovine respiratory syncytial virus (BRSV) is one of the main causes of severe pneumonia, interstitial edema, and emphysema in cattle [1]. BRSV is an RNA virus with a non-segmented, single-stranded, negative-sense 15.2 kb genome that belongs to the genus *Pneumovirus* and family *Paramyxoviridae* [2]. BRSV infection leads to sudden fever, rhinitis, cough, respiratory distress, abdominal breathing, and decreased appetite [3].

The previous epidemiological studies reported that the prevalence of BRSV in cattle ranged from 28% to 70%, depending on animal age and environmental conditions [4-6]. The disease occurs in most countries worldwide and affects cattle of all ages, with younger animals being at the greatest risk of severe BRSV disease [7,8].

Diagnosis of BRSV can be confirmed through different laboratory tests including virus isolation and antibody detection in serum and milk. Serological investigations such as serum neutralization test, complement fixation test, immunoprecipitation, and enzyme-linked immunosorbent assay (ELISA) are commonly used methods for BRSV diagnosis [9,10]. BRSV can also be accurately diagnosed using reverse transcriptase polymerase chain reaction [11].

A seroprevalence survey for BRSV in cattle has never been carried out in the Nineveh Governorate, Iraq. Therefore, this study aimed to ascertain the seroprevalence of BRSV in this region and to investigate the risk factors associated with the disease.

## Materials and Methods

### Ethical approval

Ethical approval is not necessary for such type of study. However, samples were collected as per the standard sample collection procedure without any stress or harm to the animals.

### Study area and sample size

The study was conducted between September 2017 and September 2018 in the northern, eastern, southern, and western areas of the Nineveh Governorate, Iraq. Cattle of different ages and breeds with no vaccination history against BRSV were included in the study.

The sample size was calculated according to the method of Jaykaran and Tamoghna [12], with an expected disease prevalence of 50%, confidence level of 95%, and standard error rate of 5%, using the following equation:


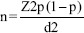


Where, n = sample size, z = confidence level, p = expected disease prevalence, and d = standard error ratio. According to the equation, 384 samples were required to conduct this study. A total of 450 blood samples were collected.

Epidemiological information (animal origin, age, sex, breed, type of breeding, season, number of cows in the herd, and geographical area) was recorded using a special examination card. The blood samples collected from the animals were processed to extract the serum and stored at −20°C until further analysis.

### Laboratory analysis

The serum samples were tested using the indirect MonoScreen AbELISA kit (Bio-X Diagnostics S.A, Belgium), according to the manufacturer’s instructions. Samples showing values ≤20% were considered negative, while those showing values between 21% and 40% were considered positive.

### Statistical analysis

Statistical analysis was performed using IBM SPSS Statistics for Windows, version 19 (IBM Corp., Armonk, N.Y., USA). The two-sided Chi-square test and Fisher’s exact test were used to assess the difference in BRSV prevalence and various risk factors in the different cattle groups. The rate of relative ratio (RR) between BRSV risk factors was calculated at 95% significance using Epi-Info TM 7, version 7 (CDC, Atlanta, GA, USA).

## Results

**Figure-1:**
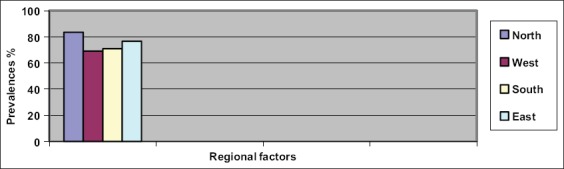
Regional factors associated with the prevalence of bovine respiratory syncytial virus.

The results of the indirect ELISA revealed that the overall prevalence of BRSV in the Nineveh Governorate was 83.11%, with the highest prevalence in cattle that were aged >7 months-1.5 years (relative risk (RR)=2.12) (Table-1). BRSV prevalence was higher in imported animals, compared to animals of a local origin (RR=1.17) (Table-1), and in animals originating from large herds (100 animals), compared to those from small herds (40 animals) (RR=1.48) (Table-1). There was no significant difference (p<0.05) between the prevalence of the disease in male and female animals (Table-1). BRSV prevalence varied significantly (p<0.05) across the different geographical areas of the Nineveh Governorate with the samples collected from the northern region displaying the highest prevalence (RR=1.33) (Figure-1). In addition, samples collected in the winter displayed the highest prevalence of BRSV (RR=1.38) compared to those collected in the spring, summer, or fall (85.09%, 83.18%, and 75.18%, respectively) (Table-2).

**Table-1:** Relative risk factors associated with the prevalence of bovine respiratory syncytial virus in cattle.

Factors	Number of cattle tested	Indirect enzyme-linked immunosorbent assay test			

		Number of positive (%)	RR	95% confidence interval	p-value
Age					
≥7 months	85	40 (47)^a^	1		
<4 years	30	15 (50)^a^	0.94	0.61-1.43	0.83
<1.5-4 years	175	159 (90.8)^b^	1.93	1.53-2.43	0.000
<7 months-1.5 years	160	160 (100)^b,c^	2.12	1.53-2.43	0.000
Gender					
Female	141	115 (81.5)^a^	1		
Male	309	259 (83.8)^a^	1.02	0.93-1.12	0.58
Origin					
Native	191	144 (75.3)^a^	1		
Imported	259	230 (88.8)^b^	1.17	1.07-1.29	0.0002
Herd size					
Small ≤10	191	124 (64.9)^a^	1		
Large ≥20	259	250 (96.5)^b^	1.48	1.33-1.65	0.000

Values significantly different (p<0.05) labeled with different letters (a, b, or c)

**Table-2:** Seasonal factors associated with the prevalence of bovine respiratory syncytial virus.

Factors	Number of cattle tested	Indirect enzyme-linked immunosorbent assay test			

		Number of positive (%)	RR	95% confidence interval	p-value
Fall	86	60 (70.5)^a^	1		
Summer	107	89 (83.1)^a,b^	1.19	1.40-1.01	0.03
Spring	156	43 (27.56)^c^	1.21	1.42-1.04	0.01
Winter	143	123 (86)^c,d^	1.23	1.41-1.05	0.003

Values significantly different (p<0.05) between factors are labeled with different letters (a, b, or c)

## Discussion

BRSV is one of the main respiratory pathogens responsible for serious economic losses to the cattle industry [13]. In this study, the overall prevalence of BRSV in the Nineveh Governorate was 83.11%; higher or lower prevalence was recorded in other countries. In Turkey, Saudi Arabia, Iran, and Norway, the reported prevalence rates were 34-96.64% [14,15], 75.5% [16,17], 51.1-100% [18,19], and 54% [20], respectively. High variation in the prevalence estimates among cattle from different countries may be due to several factors such as differences in the disease management, laboratory methods, geographical region, and disease control programs. Our study recorded the highest BRSV prevalence in animals aged between 7 and 18 months, compared to those from other age groups, which is consistent with the findings of the previous studies [21,22].

We also showed that the prevalence was significantly higher in imported animals than in domestic animals, which may be due to the stress of transportation and different environmental conditions [23]. Our finding of a higher prevalence rate in large herds compared to small ones is in agreement with those of the previous studies [24-27] and can be explained based on the fact that large numbers of animals in one farm can lead to overcrowding and close contact between animals, facilitating disease spread.

The lack of a significant difference (p<0.05) between BRSV prevalence in male and female animals was observed previously [8,28]. The significant regional variation in the disease prevalence, which was also reported in the previous studies [4,26], can be attributed to several factors including differences in the number of animals, intensity of breeding, and movement of animals. The highest BRSV prevalence in the winter months, which was also noted by Klem *et al*. [20] and Bidokhti *et al*. [21], may be caused by overcrowding, poor ventilation, and high humidity in the barns.

## Conclusion

The current report is the first epidemiological investigation of BRSV prevalence among cattle in the Nineveh Province, Iraq. Our results revealed a high prevalence of the disease in the cattle of this region and identified several risk factors associated with BRSV infection. More studies are required to further our understanding of the disease.

## Authors’ Contributions

MIA designed and planned the study. KJH and SDH did the collection of samples. KJH analyzed the samples. MIA and KJH interpreted the results. All authors read and approved the final manuscript.
